# Phytochemical Investigation, Antioxidant Properties and In Vivo Evaluation of the Toxic Effects of *Parthenium hysterophorus*

**DOI:** 10.3390/molecules27134189

**Published:** 2022-06-29

**Authors:** Javed Iqbal, Ayaz Ali Khan, Tariq Aziz, Waqar Ali, Saeed Ahmad, Shafiq Ur Rahman, Zafar Iqbal, Anas S. Dablool, Mashael W. Alruways, Abdulraheem Ali Almalki, Abdulhakeem S. Alamri, Majid Alhomrani

**Affiliations:** 1 Department of Biotechnology, Faculty of Biological Sciences, University of Malakand, Chakdara 18800, Pakistan; mailtoiqbal5544@gmail.com (J.I.); waqarali@uom.edu.pk (W.A.); 2 Pak-Austria Fachhochschule, Institute of Applied Sciences and Technology, Haripur 22621, Pakistan; 3 Department of Zoology, Faculty of Biological Sciences, University of Malakand, Chakdara 18800, Pakistan; abutalhauom@gmail.com; 4 Department of Environmental Sciences, Shaheed Benazir Bhutto University, Sheringal 18050, Pakistan; yousafzaishafiq2020@gmail.com; 5 Institute of Nursing Sciences, Khyber Medical University, Peshawar 25120, Pakistan; zafar.ins@kmu.edu.pk; 6 Department of Public Health, Health Sciences College Al-Leith, Umm Al-Qura University, Makkah al-Mukarramah 24382, Saudi Arabia; asdablool@uqu.edu.sa; 7 Department of Clinical Laboratory Sciences, College of Applied Medical Sciences, Shaqra University, Shaqra 15273, Saudi Arabia; m.alruways@su.edu.sa; 8 Department of Clinical Laboratory Sciences, The Faculty of Applied Medical Sciences, Taif University, P.O. Box 11099, Taif 21944, Saudi Arabia; almalki@tu.edu.sa (A.A.A.); a.alamri@tu.edu.sa (A.S.A.); m.alhomrani@tu.edu.sa (M.A.)

**Keywords:** *Parthenium hysterophorus*, antioxidants, HPLC, total phenolic contents, total flavonoid contents

## Abstract

*Parthenium hysterophorus L*. is a poisonous *Asteraceae* weed. The phytochemical profile, antioxidant activity, total phenolic contents (TPC), total flavonoid contents (TFC), and cytotoxicity of *Parthenium hysterophorus L*. flower extract were evaluated in this study, and the toxic effects were assessed in rabbits. The HPLC-DAD system was used for phytochemical analysis. The hemolytic and DPPH assays were performed. The effects of orally administering the flower crude extract to rabbits (n = 5) at four different doses (10, 20, 40, and 80 mg/kg) for ten days on hematological and biochemical parameters were investigated. The crude extract of the flower contained phenolic compounds such as Gallic acid, Chlorogenic acid, Ellagic acid, and P Coumaric acid, which were detected at different retention times, according to the HPLC results. With a sample peak of 4667.475 %, chlorogenic acid was abundant. At concentrations of 80 µg, the methanolic extract of flowers had total phenolic contents (89.364 ± 4.715 g GAE/g) and total flavonoid contents (65.022 ± 2.694 g QE/g). In the DPPH free radical scavenging assay, 80 µg of extract had the highest cell inhibition of 76.90% with an IC50 value of 54.278 µg/µL, while in the hemolytic assay 200 µg of extract had the highest cell inhibition of 76.90% with an IC50 > 500. The biochemical and hematological parameters were altered in the flower extract-fed groups as compared to the control (*p* < 0.05). The toxic effects on the blood, liver, and kidneys were confirmed. The findings also confirmed the presence of phenolic and flavonoid content in the flower extract, both of which contribute to the plant’s antioxidant potential.

## 1. Introduction

*Parthenium hysterophorus* is a highly invasive ruderal species annual weed that is rapidly spreading throughout Asia and other areas beyond its native range in the central region and South America and southern USA, with highly deleterious effects on native biodiversity, human and animal health, and crop and pasture productivity [1,2,3]. After 1955, the Parthenium became a crucial problem as a weed in agro habitats in more than 30 nations. *P. hysterophorus* permeates a diversity of landscapes, including farms, fallows, orchards, and train tracks [4]. The *P. hysterophorus* weed is a member of the Asteraceae family, a large and distinct species with a worldwide distribution [5]. Due to its many characteristics, such as its short life cycle of 90 to 120 days, adaptability to photothermal conditions, lack of natural enemies (generally) and rapid growth, ability to spread through waterways and roads [6], it can spread very quickly [7]. Several factors contribute to the invasion of *P.* *hysterophorus*. It can thrive under a wide range of weather conditions, germinating at temperatures ranging from 12 °C to 27 °C, tolerating saline conditions, and with a deep root that allows it to survive in areas of low humidity [8,9]. When it is young, it produces a cluster of leaves near the soil’s surface. As it matures, many branches develop on the upper half of the plant, and it grows to a height of 1.5 to 2 m [10,11].

*Parthenium* is suspected to have been spread by vehicles or as a contaminant of seed lots in many cases [2]. The propagation of its seeds depends on the supply of water, livestock, and movement of machinery. It has other common names, such as Santa Maria feverfew; bitter weed, which refers to the plant-based flowering plant; and (sometimes) carrot grass. The common name for *P. hysterophorus* L. (Asteraceae) is Skha botay in Khyber Pakhtunkhwa, Pakistan [12,13]. It is a fast-maturing annual weed with a deep root and an erect stem that becomes woody with age. Due to its allelopathic impact, this weed is believed to cause allergic respiratory problems, mutagenicity in humans and livestock, and severe reductions in agricultural production [14,15]. A research study performed by Bajwa et al. [16], demonstrated that this toxic and aggressive species is one of the worst weeds in the world. This weed is responsible for multi-million losses in Australia and is considered a harmful crop in over 45 countries [17,18]. This plant is not only harmful to agriculture but is also an important factor in various human diseases. Among these are asthma, cancer, allergies, and stomach diseases [19]. In addition, it can reduce milk production. The plant, if consumed, can cause hair loss, eye irritation, skin sores, anorexia, pruritus, alopecia, dermatitis, diarrhea, mouth ulcers with excessive salivation, and possibly death from internal tissue and organ rupture and hemorrhage [20,21,22,23,24,25].

This noxious weed pollen causes skin allergies as well as other allergic reactions, including inflammation of the eyebrow, cold reddening of the eyes, asthma, etc. This toxic plant affects humans and livestock negatively [23]. After feeding *P. hysterophorus* methanolic extract to animal models, the number of RBCs, lymphocytes, leucocytes, HB, hematocrit, WBCs, and platelets decreased significantly, while the number of neutrophils increased significantly [24,25]. According to several studies [4,19,20], the presence of these chemicals inhibits the growth and germination of a vast array of plant crop species, including native plants as well as numerous crop and pasture species. This weed contains various compounds such as sesquiterpene lactones, parthenin, quercelagetin, p-hydroxybenzene, vanillic acids, caffeic acids, p-coumaric acids, p-anisic acids, chlorogenic acids, ferulic acids [19,20].

This study reports the total phenolic and flavonoid contents in *P. hysterophorus* flowers. In addition, HPLC-DAD was used to characterize the methanolic extract of *P. hysterophorus* flowers to identify various phenolic compounds. Radical scavenging assay was performed to identify the antioxidant potential of the plant. A hemolytic assay was carried out to analyze the cytotoxic effects of the plant. The effects of methanolic flower extract on hematological, and biochemical parameters and liver and kidneys were examined.

## 2. Materials and Methods

### 2.1. Plant Collection and Identification

The *P. hysterophorous* plants were collected in September 2020 at the coordinates 34.7305N, 72.0186E in Ouch District Dir Lower Khyber Pakhtunkhwa, Pakistan. Taxonomically, the plant specimen was identified by Dr. Muhammad Nisar (Department of Botany, University of Malakand, Chakdara, Pakistan). Plant specimens were also archived in the Herbarium of the Department of Botany; the voucher and serial number are H.UOM.BG.774 and 7 October 2021, respectively. The flowers were separated and washed 2–3 times with water before being dried in the shade at 25 °C for 10 days.

### 2.2. Extraction Procedure

Flowers were ground into a fine powder using a mortar and pestle and a grinder. As a solvent, methanol (95 %) was used for flower extraction. 1 g of finely powdered flowers and 10 milliliters of methanol were combined at a ratio of 1:10. The mixture was transferred to a 250 mL flask and wrapped in aluminum foil. At 25 °C, the covered flask was kept for 24 h in a shaking incubator (Stuart Orbital Incubator S150). After incubation, the solution was kept for 10 min at 25 °C in a sonicator (Power Sonic 405, Seoul, Korea). After sonication, the solution was centrifuged at 13,000 rpm for 5 min using a Sigma 3K30 (Sigma Laborzentrifugen GmbH, Osterode am Harz, Germany) centrifuge to collect the supernatant. The supernatant was filtered and dried for 72 h in an open area with a fan in a petri dish. We obtained a concentrated flower crude extract (FCe) after evaporation and stored it at 4 °C for future use.

### 2.3. Phytochemical Analysis

Various studies have demonstrated that *P. hysterophorous* contain higher amounts of flavonoids and phenolic compounds in their chemical constituents [26]. The *P. hysterophorous* extracts gives better antimicrobial activity. To investigate different bioactive compounds in the FCe, the standard protocol of Ovias et al. [27] was followed using High-Performance Liquid Chromatography System, (HPLC system, Agilent 1260, Santa Clara, CA, USA). The compounds were separated using an Agilent Zorbax Eclipse C18 column. The compounds were eluted using two solvents: solvent A (methanol: acetic acid: deionized water, 90:2:8, *v*/*v*) and solvent B (methanol: acetic acid: deionized water, 90:2:8, *v*/*v*).

### 2.4. Total Phenolic Content (TPC) Determination

The Ciocalteu method was used to determine the TPC in FCe [28]. 4 mg/mL FCe in DMSO was used as a stock solution. A 10-fold diluted Folin-Ciocalteu reagent, a 6% solution of Sodium Carbonate (Na2CO_3_) (6 g/mL), and a 4 mg/mL Gallic acid (C_7_H_6_O_5_) stock solution in methanol were prepared. 20 µL FCe was placed in a 96 well plate. Using a micropipette, 90 µL of Folin-Ciocalteu reagent, was added to the sample’s well. For 5 min, the plate was incubated. 90 µL Na_2_CO_3_ was added to the sample well and properly mixed with a micropipette. Negative and positive controls were used, respectively, with DMSO and gallic acid (20 µL). At 37 °C, the plate was incubated for 90 min. A microplate reader was used to record the readings at a wavelength of 630 nm (ELx800 Bio-Tek, BioTek^®^ Instruments, Inc., Winooski, VT, USA). The experiment was repeated three times.

### 2.5. Total Flavonoid Content (TFC) Determination

The total flavonoid content was determined using the AlCl_3_ method (Aluminum trichloride) as described by [29,30]. FCe 4 mg in 1 mL DMSO, 10 gm aluminum chloride in 100 mL distilled water (dH_2_O), 1M potassium acetate in distilled water (98.15 g/100 mL), and 1 mg quercetin in 1 mL methanol were used to make the stock solution. A 96 well plate was filled with 20 µL of FCe. With the help of a micro-pipette, 10 µL of 10% aluminum chloride was added to FCe in a 96 well plate and mixed. 10 µL potassium acetate (1 M) was added. Finally, 160 µL dH_2_O was added to each well, resulting in a final concentration of 20 µL in each well, which was thoroughly mixed. Negative control of 20 μL methanol was used, while a positive control of 50, 25, 20, 10, 5, and 2.5 µg/mL Quercetin was used. The plate was incubated at 37 °C for 39 min after mixing. A microplate reader (ELx800 Bio-Tek, BioTek^®^ Instruments, Inc., Winooski, VT, USA) was used to read the samples at 415 nm. Quercetin equivalent µL/mg was used to calculate the results. Three times the experiment was repeated.

### 2.6. DDPH Assay

The extracts’ ability to scavenge DPPH (2, 2-diphenyl-1-picrylhydrazyl) free radicals was determined using the standard procedure described by Netala et al. [31] and Khan et al [32]. In this free scavenging assay, DPPH was used as the substrate. 4 mg FCe in 1 mL DMSO, 9.6 mg/100 mL DDPH in methanol, and 4 mg/mL ascorbic acid in methanol were used to make the stock solution. FCe samples of 5 µL, 10 µL, 15 µL, and 20 µL were placed in 96 well plates. Then, to acquire a final concentration of 200 µL well, 195 µL, 190 µL, 185 µL, and 180 µL of DDPH reagent were added to the sample’s well. Negative controls included different concentrations of DMSO (20, 15, 10, and 5 µL) and positive controls included 20, 15, 10, and 5 µL of ascorbic acid. Then it was incubated at room temperature for 60 min. The entire procedure took place in a dark room. A microplate reader was used to take readings at 517 nm wavelength (ELx800 BioTek, BioTek^®^ Instruments, Inc., Winooski, VT, USA). The ascorbic acid Equivalent [28] µg/mg of extract was used to calculate the IC50 values.

The percent free radical scavenging of DPPH was calculated using the formula:

$$\%\;scavenging=1-\frac{sample\;of\;absorbance}{control\;absorbance}\times 100$$

Calculated as ascorbic acid equivalent µg/mg of extract.

### 2.7. Hemolytic Assay

Hemolytic activity of the FCe was determined based on the protocol outlined [32,33,34]. For this test, erythrocytes were extracted from fresh human blood. As an anticoagulant, the blood was collected in Ethylene Diamine Tetra Acetic acid (EDTA). Blood was transferred to an Eppendorf tube and centrifuged at 14,000 rpm for five minutes. Following centrifugation, the supernatant was disposed of. The pellet was washed by adding 980 µL of Phosphate Buffer Saline (PBS) to 20 µL of pellet in Eppendorf tubes and centrifuging for 10 min. The discarding of supernatant was repeated three times. Added 4% pellet, i.e., for 5000 µL, we dissolved 200 µL of pellet in 4800 µL PBS based on the sample, and the erythrocytes suspension was ready for use. The samples (50, 40, 30, and 20 µL) were taken from a 4 mg/mL stock solution in Eppendorf tubes. Then, 150, 160, 170, and 180 µL of erythrocytes suspension were added to each tube and incubated for three hours at 37 °C. After incubation, the tubes were centrifuged at 2500 rpm for 10 min. Then 100 µL of supernatant was added to 96 well plates, and hemoglobin release was measured using a microplate reader at 540 nm wavelength (ELx800 BioTek, BioTek^®^ Instruments, Inc., Winooski, VT, USA). Positive control consisted of 50 µL of 0.55 Triton X-100, while negative control consisted of 50 µL of DMSO. The absorbance of wells containing RBCs lysed with 0.5% Triton X-100 was interpreted as 100 percent hemolysis. The percentage of hemolysis was calculated using the following formula:

$$\%\;hemolysis=\frac{O.D\;of\;sample-O.D\;in\;PBS}{O.D\;in\;0.5\%\;Triton\;X-100-O.D\;in\;PBS}\times 100$$

### 2.8. In Vivo Toxic Potential of Methanolic Extract of Flower

Local Himalayan rabbits were purchased in Chakdara, Dir Lower, Khyber Pakhtunkhwa, Pakistan, and kept in the animal house of the University of Malakand’s Department of Biotechnology. Green leaves and freshwater were freely available to the rabbits. The experiment and animal handling were carried out in accordance with the ARRIVE guidelines of the Pakistan Ethics Committee for Animal Care and Use. The study was approved by the University of Malakand’s Department of Biotechnology’s ethical committee. The rabbits were divided into different groups before the experiment began. There were three rabbits in each group. FCe was given to group A at a dose of 10 mg/kg body weight, group B at a dose of 20 mg/kg body weight, group C at a dose of 40 mg/kg body weight, and group D at an 80 mg/kg body weight. The FCe was provided on a 10-day basis. A normal diet and water were provided to the control group.

### 2.9. Blood Collection and Analysis

On the tenth day of the experiment, blood samples (about 5 mL) from the jugular vein were collected from all groups in EDTA tubes for whole blood collection, and gel tubes for serum isolation. Strict aseptic conditions were observed during blood sampling. The automatic digital machine (Sysmex Kx-21, Sysmex Corporation, Kobe, Japan) was used to determine hematological parameters from whole blood. The ultraviolet-visible spectrometer (UV-VS) (T60, PG instrument, Leicestershire, UK) was used to measure biochemical parameters in serum.

### 2.10. Statistical Analysis

The obtained results were statistically analyzed. All the parameters’ data were expressed as mean standard deviation [35] and analyzed using one-way analysis of variance (ANOVA), followed by Tucky multiple comparison tests. A *p*-value less than or equal to 0.05 was considered statistically significant. For statistical analysis, the online software Graph Pad Prism Demo version 5 was utilized (www.graphad.com, accessed on 24 November 2021).

## 3. Results

### 3.1. Characterization of FCe

**Figure 1:**
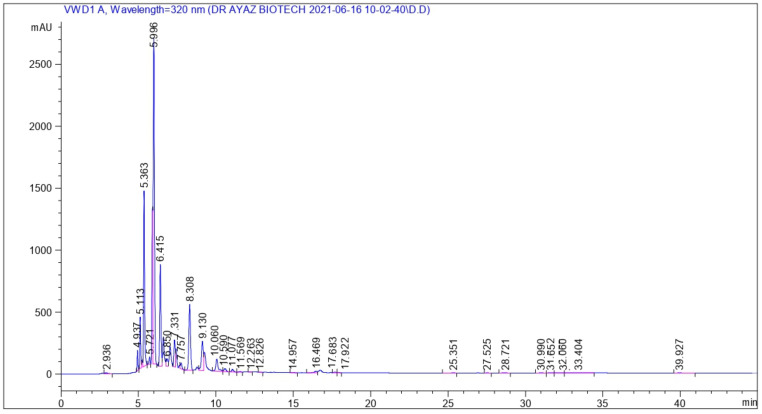
The HPLC chromatogram of flower methanolic extract of *P*. *hysterophorous*.

For the identification of various bioactive compounds, HPLC analysis was performed on FCe. Figure 1 depicts the retention time and peak area of various phenolic compounds in accordance with available references and standards. At a retention time of 2.61 min and a peak area of 113.83, Malic acid was identified. Gallic acid, Chlorogenic acid, Epigallocatechin gallate, Bis-HHDP-hex(pedunculagin), Morin, Ellagic acid, Kaempferol-3-(caffeoyl-diglucoside)-7-rhamnosyl, Kaempferol-3-(p-coumaroyl diglucoside)-7-glucoside, Pyrogallol, Mandelic acid, Quercetin-3 (caffeoyldiglucoside)-7-glucoside, p-coumaric acid were detected at retention times of 4.93, 6.41, 8.30, 11.59, 12.26, 16.46, 25.35, 27.52, 28.72, 30.99, 31.65, and 32.06 min, as shown in Table 1.

The phenolic content of the methanolic extract of the flower of *P. hysterophorus* was 89,364 ± 4715 g GAE/g of extract, and the total flavonoid content was 65,022 ± 2694 g QE/g of extract.

### 3.2. DPPH Assay

**Figure 2:**
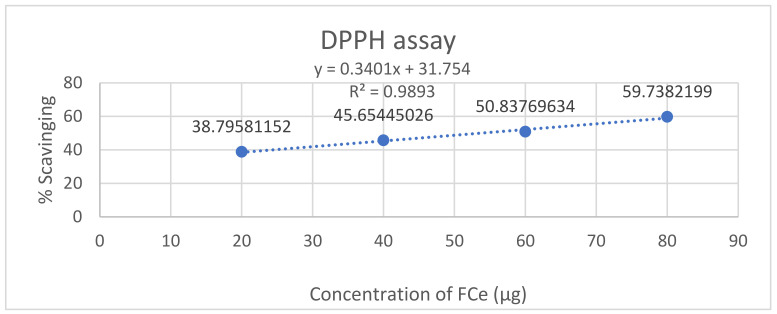
DPPH free radical scavenging activity of flower methanolic extract of *P. hysterophorus.*

The antioxidant potential of FCe was evaluated using the DPPH assay at concentrations of 20, 40, 60, and 80 µg. As the concentration of FCe increased, the percentage of scavenging increased gradually, with an IC50 value of 54.27 µg AAE/g of extract. At a concentration of 80 µg, it was observed that the crude extract possesses the highest free radical scavenging activity with 59.73 percent inhibition, followed by 60, 40, and 20 µg of the flower’s crude extract with scavenging activities of 50.83, 45.65, and 38.79 percent, respectively. Ascorbic acid was used as the standard. The percentage of DPPH radical scavenging of FCe has been given in Table 2 while DPPH free radical scavenging activity of flower methanolic extract of *P. hysterophorus* is shown in Figure 2.

### 3.3. Hemolytic Assay

**Figure 3:**
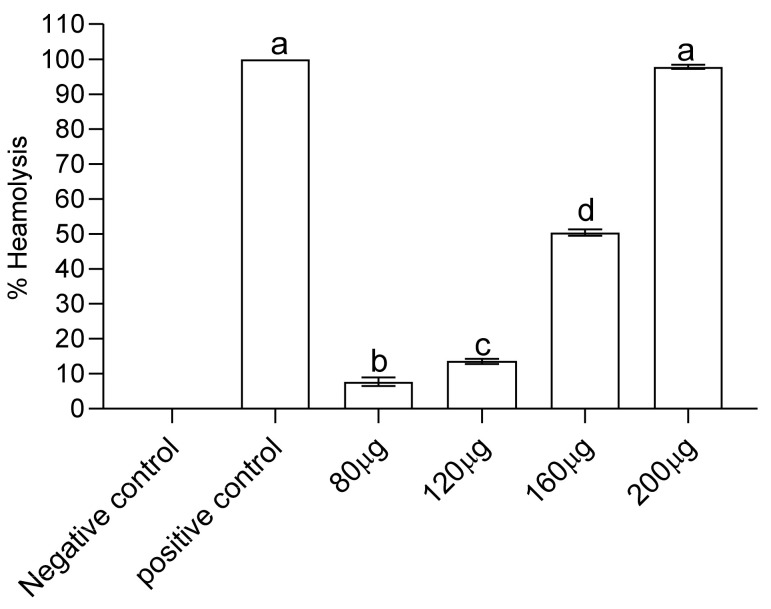
Hemolytic activity of flower methanolic extract of *P. hysterophorus.* Different alphabets (a, b, c & d) on bar graph show significant difference while same alphabets show no significant difference (*p* < 0.05).

FCe’s hemolytic activity was evaluated using fresh human erythrocytes to determine its potential hemolytic activity. FCe activity was measured as a percentage of hemolysis. FCe lysed 7.691, 13.530, 50.360, and 97.790 percent of cells with IC50 > 500 at concentrations of 80, 120, 160, and 200 µg/ml. BPS + erythrocytes were used as a negative control, resulting in 0% hemolysis, while Triton X-100 was used as a positive control, resulting in 100% hemolysis. Except for 80 µg, which had only 7.691 percent cell lyses, all concentrations caused hemolysis. The hemolysis was found to be directly proportional to the FCe dose as shown in Figure 3.

### 3.4. Analysis of Hematological Parameters

Compared to the control, administration of flower methanolic extract of *P. hysterophorus* (10 mg/kg, 20 mg/kg, 40 mg/kg, 80 mg/kg body weight) significantly decreased the number of white blood cells, red blood cells, and platelets in rabbits. A dose-dependent and statistically significant increase in Hb, MCV, MCH, MCHC, MPV, and PDW values was also observed in rabbits given a methanolic extract of the flowers of *P. hysterophorus*. Additionally, the extract decreased the number of PLT, PCT, neutrophils, lymphocytes, and monocytes. In comparison to the control group, the decrease was statistically significant (*p* < 0.05) across all groups. The results are given in Table 3.

### 3.5. Serum Biochemical Parameters

The effects of administering a methanolic extract of the flowers of *P. hysterophorus* on the biochemical parameters of rabbits are shown in Table 4. Compared to the control group, administration of FCe to rabbits significantly increased (*p* < 0.05) ALP, ALT, blood urea, and serum creatinine while significantly decreasing (*p* < 0.05) serum cholesterol, triglycerides, HDL, and LDL. Serum bilirubin levels were not significantly different (*p* < 0.05) between the FCe-fed and control groups.

## 4. Discussion

In the present study, we sought to determine the phytochemical composition, hemolytic activity, radical scavenging abilities, and in vivo toxic effects of *P. hysterophorus* flower methanolic extract. FCe was subjected to qualitative phytochemical analysis, which revealed the presence of alkaloids, flavonoids, terpenoids, chlorophyll, and phenols. The FCe was then analyzed with HPLC to identify its bioactive compounds.

The phytochemical composition of various plants is responsible for the biological activities observed in them. As a result, their phenolic and flavonoid content are two important components for biological activities such as antioxidant potential [32,33,34,35,36,37,38]. Furthermore, at 80 µg, FCe contained high levels of flavonoids and phenolics (65.022 g QE/g and 89.364 g GAE/g). The antioxidant activity was also high at 80 µg of FCe, demonstrating the remarkable relationship between phenolic and flavonoid contents and percent DPPH scavenging reported by [38,39]. Different studies found the link between phenolic content and antioxidant activity [40,41]. The presence of high phenolic and flavonoid content can be attributed to the FCe’s antioxidant abilities. In vitro testing of the antioxidant activity of biological materials is carried out with DPPH, a synthetic compound. Once their solutions have been incubated, they produce free radicals. In their oxidized state, they have distinct colors. If the material being tested has antioxidant properties, it will donate electrons to these free radicals, causing the solution’s color to change. The percent inhibition is calculated using a UV-visible spectrophotometer to quantify the color change or decrease in color intensity [42].

The IC50 value is the most used parameter for determining free radical scavenging activity. A higher IC50 value indicates lower antioxidant activity and vice versa [43]. Significant DPPH scavenging was observed when ascorbic acid was used as a positive control. When compared to Ascorbic acid, FCe had the highest percent scavenging. The percent inhibition of free radicals increased in accordance with the increase in FCe concentration. The highest inhibition was found at 80 µg of all the concentrations. Numerous plant extracts have been evaluated for their hemolytic properties and correlated with their constituents, such as the polyphenolic antioxidants found in green and black tea [44]. If the percentage of hemolysis is greater than 30%, plant extracts are considered toxic to red blood cells [45]. At a concentration of 200 µg of FCe, 97,790,5975 percent of cells were observed to lyse with an IC50 > 500, which may be attributable to the presence of Saponins in the flowers. Hemolysis of erythrocytes appears to be caused by Saponin’s ability to form complexes with cell membrane cholesterol, resulting in pore formation, increased cell permeability, and modifications in the negatively charged carbohydrate portions on the cell surface [46].

Since blood is the body’s primary carrier of substances, its components are extremely sensitive to toxins; thus, hematological parameters represent an important clinical response to toxic compounds [47]. When compared to the control group, the difference in hematological parameters such as hemoglobin, WBCs, RBCs, and neutrophils was significantly (*p* < 0.05). Normal animal physiology requires a sufficient percentage of hemoglobin, which is determined by the erythrocyte count. The presence of Saponins in the FCe may cause hemolysis due to contact with cholesterol molecules found on the membrane of erythrocytes, which causes cell rupture. The drop in RBC levels could be attributed to the presence of Pyrogallol in FCe, which causes oxidative stress and erythrocyte membrane damage, resulting in red blood cell loss [48]. The decrease in WBC count indicates that the immune system may become weakened following FCe treatment. The platelet count was significantly reduced in the current study. FCe administration resulted in a concentration-dependent and statistically significant (*p* < 0.05) decrease in platelet count. Platelet count reduction in experimental animals has been reported to have a negative effect on the oxygen-carrying capacity of the blood as well as thrombopoietin [49].

The decrease in platelet counts observed in this study suggests that the administration of FCe may disrupt the blood’s oxygen-carrying capacity. The presence of Ellagic acid in the FCe may cause a significant change in MCV levels [50]. FCe administration to rabbits increased the levels of alanine amino transferase (ALT) or serum glutamic pyruvic transaminase (SGPT), serum alkaline phosphatase (ALP), serum urea, and serum creatinine significantly. Plasma urea and creatinine levels have been shown in human and animal studies to be useful indicators of renal function. An increase in plasma levels indicates that renal function is deteriorating [51]. Intoxication with *P.* *hysterophorus* increases the levels of liver enzymes ALT and ALP, which could be due to the generation of reactive oxygen species in the liver, which increases lipid peroxidation and toxic aldehydes, causing inflammation and necrosis [52]. Parthenin, which is found in FCe, is highly cytotoxic to kidney cells, inhibiting the synthesis of RNA, DNA, and key cellular enzymes within 24 h of exposure [52].

Lee et al. [53] proposed that the inhibition of macromolecular synthesis could be attributed to its ability to alkylate with DNA and/or inhibit nucleic acid polymerases with SH-groups at their active sites. Serum Cholesterol, Triglycerides, High-density lipoprotein (HDL), and Low-density lipoprotein (LDL) were all significantly lower in rabbits who were given parthenium flowers extract (LDL). The presence of p-Coumaric acid in FCe, which activates brown adipose tissue, could explain the significant decrease (*p* < 0.05) in lipids. This was mediated by the mammalian target of rapamycin complex 1 (mTORC1)-RPS6 pathway, which resulted in increased energy expenditure and thermogenesis [54].

## 5. Conclusions

The methanolic extract of *Parthenium hysterophorus* flowers was found to contain phenolic and flavonoid compounds. Flower extract showed a dose-dependent cytotoxic effect. To inhibit radicals, the FCe has a strong antioxidant activity. In rabbits, a methanolic extract of *P.* *hysterophorus* flowers has a toxic effect on hematological and biochemical parameters, i.e., blood, kidneys, and liver.

*P. hysterophorus* has abundant biological applications, but still its full potential has not been fully exploited and scientific data is limited. In addition, the commercial application of *P. hysterophorus* for the manufacturing of drugs to cure different diseases is an area of study that needs to be investigated. *P. hysterophorus* gives an excellent source of bioactive molecules, including Sesquiterpene lactones (SLs), which have vital medicinal properties.

## Figures and Tables

**Table 1:** Identification of phenolic compounds through HPLC-DAD in flower methanolic extract of *P. hysterophorous*.

Retention Time (min)	Phytochemical Compounds	HPLC-UV λmax (nm)	Sample Peak Area	Area %	Identification Reference
2.681	Malic acid	320	113.830	0.285	Ref. Stand
4.937	Gallic acid	320	574.438	1.442	Ref. Stand
6.415	Chlorogenic acid	320	467.475	11.722	Ref. Stand
8.308	Epigallocatechin gallate	320	401.196	10.081	Ref. Stand
11.596	Bis-HHDP-hex(pedunculagin)	320	37.707	0.094	[35]
12.263	Morin	320	27.504	0.069	Ref. Stand
16.469	Ellagic acid	320	177.966	0.447	Ref. Stand
25.351	Kaempferol-3-(caffeoyl-diglucoside)-7-rhamnosyl	320	68.127	0.171	[36]
27.525	Kaempferol-3-(p-coumaroyl-diglucoside)-7-glucoside	320	28.542	0.071	[36]
28.721	Pyrogallol	320	60.840	0.152	Ref. Stand
30.990	Mandelic acid	320	59.972	0.150	Ref. Stand
31.652	Quercetin-3-(caffeoyldiglucoside)-7-glucoside	320	53.121	0.133	[37]
32.060	p-Coumaric acid	320	79.998	0.200	[37]

**Table 2:** The percent DPPH free radical scavenging activity of the FCe using ascorbic acid as the standard.

Sample	Concentration (µg)	DPPH % Inhibition	DPPH IC50 (µg AAE/ µg)
FCe	80	59.73	54.278
	60	50.83	
	40	45.65	
	20	38.79	

**Table 3:** Blood hematological parameters of control and groups fed with flower methanolic extract of *P. hysterophorus*.

Parameters	Control	A	B	C	D
WBC (×10^6^ /µL)	9.704 a	8.73 ± 0.105 b	7.7 ± 0.215 c	6.63 ± 0.334 d	5.53 ± 0.150 e
RBC (×10^6^ /µL)	6.801 a	6.17 ± 0.155 a	5.35 ± 0.251 b	5.1 ± 0.524 b	3.94 ± 0.240 c
HGB (mg/dL)	10.002 a	10.70 ± 0.204 b	11.13 ± 0.205 b	11.80 ± 0.263 c	12.5 ± 0.361 d
HCT (%)	37.405 a	34.97 ± 0.752 b	31.27 ± 1.114 c	29.47 ± 0.656 c	27.23 ± 0.905 d
MCV (pg)	70.502 a	67.43 ± 0.906 b	63.27 ± 0.420 c	60.80 ± 0.883 d	58.50 ± 0.781 e
MCH (pg)	18.806 a	19.50 ± 0.362 a	21.17 ± 0.902 c	23.40 ± 0.881 d	24.30 ± 1.154 d
MCHC (mg/dL)	26.606 a	28.57 ± 0.56 b	29.30 ± 0.224 c	29.77 ± 0.325 d	35.73 ± 0.410 c
PLT (×10^3^ /µL)	241.024 a	148.7 ± 2.081 b	140.3 ± 1.524 c	132.3 ± 1.526 d	115.3 ± 2.151 e
RDW (%	20.403 a	22.33 ± 1.052 a	25.37 ± 1.150 b	29.47 ± 0.852 c	32.07 ± 0.354 d
PCT (ng/ml	0.164 a	0.11 ± 0.015 a	0.8 ± 0.161 b	0.6 ± 0.100 b	0.3 ± 0.125 a
MPV (fL)	6.701 a	6.40 ± 0.132 a	6.06 ± 0.252 a	4.63 ± 0.458 b	3.46 ± 0.253 c
PDW (fL)	15.605 a	16.37 ± 0.158 b	17.1 ± 0.260 c	18.2 ± 0.306 d	19.07 ± 0.150 e
Neutrophils (%)	64.001 a	62.00 ± 1.085 a	60.33 ± 0.571 a	56.67 ± 1.526 b	54 ± 1.061 c
Lymphocytes (%)	32.004 a	30.33 ± 0.572 a	28.67 ± 1.521 a	25.33 ± 0.570 b	22.67 ± 1.528 b
Monocytes (%)	3.004 a	2.66 ± 0.576 a	2.0 ± 0.017 a	1.33 ± 0.571 b	1.0 ± 0.086 b

The same alphabets in a row show no significant difference (*p* < 0.005), while different alphabets in a row show significant difference (*p* < 0.005). A = group fed with FCe at dose rate of 10 mg/kg, B = group fed with FCe at dose rate of 20 mg/kg, C = group fed with FCe at dose rate of 40 mg/kg, D = group fed with FCe at dose rate of 80 mg/kg.

**Table 4:** Blood biochemical parameters of control and groups fed with flower methanolic extract of *P. hysterophorus*.

Parameters	Control	A	B	C	D
ALT (U/L)	34.018 ± 1.144 a	52.036 ± 3.017 b	68.672 ± 3.214 c	86.331 ± 3.054 d	92.702 ± 2.007 e
Bilirubin (mg/dL)	0.704 ± 0.337 a	0.464 ± 0.157 a	0.734 ± 0.155 a	0.706 ± 0.264 a	0.903 ± 0.114 b
ALP (IU/L)	219.016 ± 3.042 a	239.041 ± 2.055 b	254.713 ± 1.524 c	276.015 ± 3.641 d	295.051 ± 2.017 e
Blood urea (mg/dL)	22.091 ± 1.2 a	36.677 ± 2.051 b	56.674 ± 2.515 c	76.331 ± 1.527 d	83.673 ± 1.541 e
Creatinine (mg/dL)	0.927 ± 0.117 a	0.933 ± 0.256 a	1.207 ± 0.104 a	1.529 ± 0.144 b	1.663 ± 0.157 b
Cholesterol (mg/dL)	156.012 ± 3.209 a	137.049 ± 1.008 b	129.040 ± 1.058 c	129.311 ± 2.507 c	110.725 ± 2.086 d
Triglycerides (mg/dL)	131.004 ± 1.532 a	128.717 ± 1.526 a	119.015 ± 2.029b	112.318 ± 1.524 c	107.317 ± 0.572 c
HDL (mg/dL	55.017 ± 2.163 a	53.334 ± 1.522 a	49.671 ± 1.155b	48.330 ± 1.521 b	46.336 ± 0.575 c
LDL (mg/dL)	81.004 ± 1.850 a	74.673 ± 1.528 b	66.671 ± 1.529 c	59.672 ± 0.570 d	46.144 ± 1.041 e

The same alphabets in a row show no significant difference (*p* < 0.005), while different alphabets in a row show significant difference (*p* < 0.005). A = group fed with FCe at dose rate of 10 mg/kg, B = group fed with FCe at dose rate of 20 mg/kg, C = group fed with FCe at dose rate of 40 mg/kg, D = group fed with FCe at dose rate of 80 mg/kg.

## Data Availability

Not applicable.
